# Oral mRNA Vaccines Against Infectious Diseases- A Bacterial Perspective [Invited]

**DOI:** 10.3389/fimmu.2022.884862

**Published:** 2022-05-03

**Authors:** Vijayakumar Jawalagatti, Perumalraja Kirthika, John Hwa Lee

**Affiliations:** Department of Veterinary Public Health, College of Veterinary Medicine, Jeonbuk National University, Iksan, South Korea

**Keywords:** bacterial delivery, alphaviral replicon, mRNA vaccine, oral, mucosal vaccine, SARS-CoV-2, infectious diseases

## Abstract

The mRNA vaccines from Pfizer/BioNTech and Moderna were granted emergency approval in record time in the history of vaccinology and played an instrumental role in limiting the pandemic caused by SARS-CoV-2. The success of these vaccines resulted from over 3 decades of research from many scientists. However, the development of orally administrable mRNA vaccine development is surprisingly underexplored. Our group specializing in *Salmonella*-based vaccines explored the possibility of oral mRNA vaccine development. Oral delivery was made possible by the exploitation of the Semliki Forest viral replicon and *Salmonella* vehicle for transgene amplification and gene delivery, respectively. Herein we highlight the prospect of developing oral replicon-based mRNA vaccines against infectious diseases based on our recent primary studies on SARS-CoV-2. Further, we discuss the potential advantages and limitations of bacterial gene delivery.

## Introduction

Edward Jenner’s innovative contribution played a pivotal role in the ultimate eradication of smallpox and served as the harbinger of vaccination. This was followed by the works of Louis Pasteur, who spearheaded the development of live-attenuated cholera vaccine and inactivated anthrax vaccine in humans in 1897 and 1904, respectively. The field of vaccine research soon became popular, and vaccines were developed against a plethora of infectious diseases of medical and veterinary importance. First-generation traditional vaccines based on the use of live, live-attenuated, and inactivated organisms were instrumental in the control of measles, polio, rubella, mumps, classical swine fever, and many other diseases, and responsible for the eradication of smallpox in humans and rinderpest in cattle. Although live and live-attenuated vaccines are effective, they may pose significant health risks to vaccinated individuals, including the development of disease, transmission to healthy individuals, and reversion to a virulent form and particularly in individuals with compromised immune system (1–5). All this changed with the advent of molecular biology and recombinant DNA technology, which paved the way for the development of safer vaccines. However, DNA vaccines did not achieve their expected clinical success owing to limited or poor immunogenicity (6, 7). Technological refinements were made to improve DNA vaccine efficacy (8–15), but the risk of mutagenesis induced by exogenous DNA integration has limited their use in humans (16–19). This has led to a renewed interest in the use of RNA in vaccines and therapeutics.

Synthetic RNA vaccines fall into two main categories: non-replicating and self-amplifying mRNA vaccines. The non-replicating mRNA vaccine is a straightforward approach wherein administered mRNA is directly translated in the cytoplasm of transfected cells to produce immunogenic proteins. The extent of non-replicating mRNA vaccine-induced antigen expression is proportional to the number of transfected cells and thus, requires the injection of a large dose of mRNA. This can be overcome by the use of self-amplifying RNA replicons from alphaviruses, such as Sindbis virus (20), Semliki Forest virus (SFV) (21), and Venezuelan equine encephalitis virus (VEE) (22). Different vector systems, namely replication-competent viral particles, replication-deficient viral particles, and DNA-launched-mRNA vector approaches, have been exploited for transgene expression (reviewed in 23, 24). DNA-launched-mRNA vectors were engineered by deleting the structural genes from the genome and replacing them with the target genes (21, 25). The resulting vector backbone with non-structural proteins (nsp1–4) forms a replicase complex that drives efficient transgene expression by a self-amplifying mechanism (21, 24). The mRNA vaccines developed to combat SARS-CoV-2 constitute the first success story in the long history of mRNA vaccine development. Nonetheless, oral delivery of an mRNA vaccine has surprisingly not been exploited. In this article, we highlight a strategy for the development of oral replicon-based mRNA vaccines by taking cues from our recent publications and discussing the advantages of *Salmonella*-mediated oral gene delivery.

## mRNA Vaccines: A Brief Historical Background

**Figure 1:**
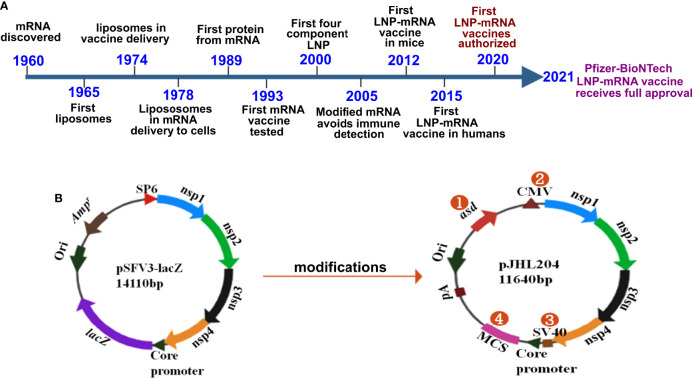
History and design of mRNA vaccines. **(A)** Timeline depicting some of the key milestones that contributed to the first successful mRNA vaccines developed against COVID-19. The timeline was adapted from Sahin et al., 2014 (ref. 27) Hou et al., 2021 (28); and Dolgin, 2021 (29). **(B)** The DNA-launched-mRNA vaccine design for bacterial delivery. pSFV3-lacZ, an SFV replicon-based vector, was used after making several modifications. 1- The ampicillin resistance marker was replaced with asd, an auxotrophic marker to enable antibiotic-free maintenance and delivery of the vector. 2- The SP6 promoter was replaced with the cytomegalovirus (CMV) promoter. 3- The SV40 promoter was placed just before the SFV sub-genomic core promoter to enable direct nuclear transcription of the vaccine constructs. 4- *lacZ* was replaced with multiple cloning site (MCS) sequences. nsp 1-4 from SFV constitute the replicon, which drives efficient transgene expression through RdRp. pA, polyadenylation signal; Ori, pBR origin of replication; RdRp, RNA dependent RNA polymerase; nsp, non-structural protein; SFV, Semliki Forest virus.

The vaccines developed against SARS-CoV-2 by Pfizer/BioNTech and Moderna constitute the first success stories in mRNA vaccine history. Although the delivery of mRNA wrapped in cationic liposomes was shown to produce proteins in human cells in 1989 (26), the potential of mRNA as a vaccine has yet to be exploited. During these past 3 decades, many scientists studied mRNA, and collective scientific advances enabled the production of the first successful mRNA vaccine in record time (**Figure 1A**). Some of the most important inventions to the adaptation of mRNA vaccination were the chemical modification of mRNA and lipid nanoparticles for delivery. Without lipid nanoparticle encapsulation, administered mRNA would be detected by the immune system and probably degraded by RNases. Of note, mRNA was shown to elicit TLR3-mediated immune activation of dendritic cells (DCs) (30), and bacterial RNA can prime DCs for higher IL-12 secretion (31). Replacing uridine with pseudouridine, the chemical modification that diminished immune recognition of administered mRNA, paved the way for mRNA treatments (32). The encapsulation of mRNA by lipid nanoparticles (LNPs) provided an effective and safe delivery platform (reviewed in 33). The discovery of increased protein expression and potent antibody responses to the SARS-CoV-2 spike protein in its stabilized prefusion conformation (34) is vital to the efficacy of mRNA vaccines. The developments and progress in mRNA vaccines against infectious diseases have been reviewed elsewhere (35, 36).

## mRNA Delivery Technologies: Progress and Limitations

The poor uptake of mRNA by cells is associated with the rapid degradation of naked mRNA by extracellular RNAses (37–41). Developments of efficient mRNA delivery platforms have been fruitful in the last decade. From advancements in transfection reagents and liposomes to nanoparticles and nanoemulsions, *in vivo* antigen presentation and the immune response to mRNA-based vaccines have recently improved (42–51). The aforementioned mRNA complexing strategies have been shown to affect mRNA stability during storage (52). Thus, a continuous supply of raw materials is crucial for the uninterrupted production of mRNA vaccines. Such requirements can prove challenging at times when the demand is high (52–57). Additionally, substituting rare codons with frequently used synonymous codons and introducing modified nucleosides have been shown to enhance mRNA translation and stability in the context of vaccination (reviewed in 38). A major disadvantage associated with base modifications is that they may result in altered mRNA secondary structure, which may influence translation and protein folding. These alterations may, in turn, prove detrimental to efficacy (58–60). One of the major drawbacks with *in vitro* transcribed RNA is the presence of dsRNAs that trigger the innate immune response and reduces the vaccine efficacy. Advancement such as cellulose-based purification was shown to remove the dsRNA byproducts leading to the lower type I interferon response and improving the efficacy of a self-amplifying mRNA vaccine against Zika virus (61). Continuous efforts have been made to minimize the drawbacks associated with mRNA vaccines, enabling an array of these vaccines to enter phase IIb clinical trials (38, 62–67). Most of the current mRNA vaccines against infectious disease are administered using the conventional delivery routes, namely intramuscular, subcutaneous, intradermal, or intranodal routes. Most of these routes of administration require injection and specific conditions for storage and transport. Furthermore, the concerns associated with the stability of these vaccines and the addition of adjuvants to enhance immunogenicity increase the cost of production and pose toxicity threats (68, 69). Despite the success of mRNA vaccines in controlling infectious diseases, the limitations associated with their production and administration demonstrate the need to develop better and safer routes of administration for mRNA vaccines (70, 71).

## Is It Possible to Orally Deliver mRNA Vaccines?

**Figure 2:**
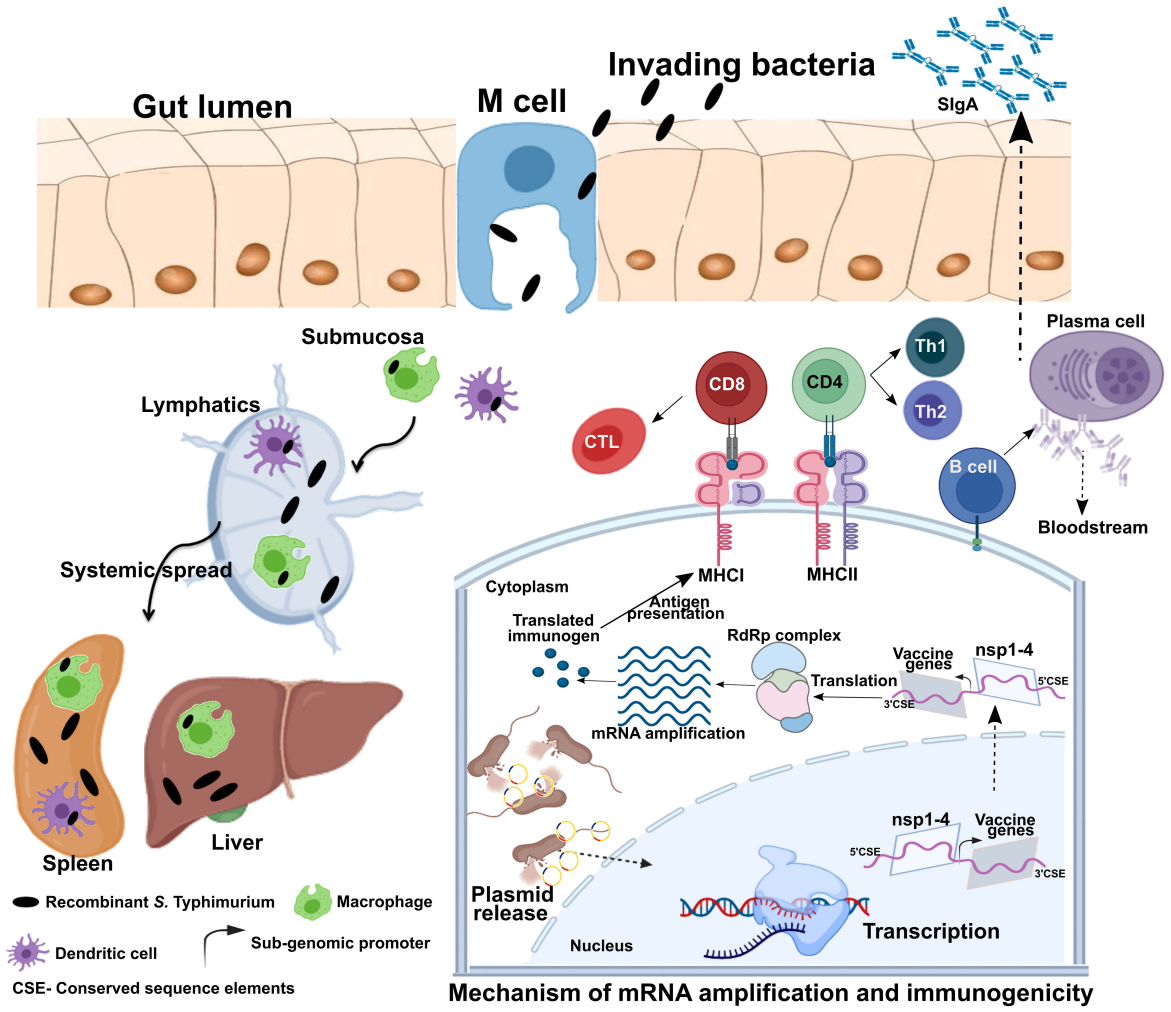
Mechanism of gene delivery, transgene expression, and induction of immune response. Upon oral administration, *Salmonella* Typhimurium is translocated from the luminal surface to submucosa by specialized M cells in the gut epithelium. Bacteria then invade antigen-presenting cells (APCs), such as macrophages and dendritic cells (DCs), and spread to different organs like the liver and spleen through lymphatics and the bloodstream. The vector encoding the Semliki Forest virus (SFV) replicon (nsp1-4) and SARS-CoV-2 immunogens is released within the host cell cytoplasm through bacterial lysis. Transcription of the delivered plasmid takes place in the cell nucleus, and, following *in situ* translation, the nsp1-4 proteins form an RNA-dependent RNA polymerase (RdRp) complex. The RdRp complex then recognizes the sub-genomic promoter and flanking conserved sequence elements (CSE), leading to enhanced mRNA amplification of vaccine genes. The resulting mRNAs translated to produce immunogenic proteins. The APCs process and present antigens to CD8+ and CD4+ T cells *via* MHC I and MHC II, respectively, leading to the elicitation of the T cell response. DCs can present antigens directly to B cells or follicular DCs (FDCs). FDC stores antigens for a longer time, periodically displaying them to cognate B cells. B cells then differentiate to specific antibody-secreting plasma cells and memory B cells. MHC, major histocompatibility complex; nsp, non-structural protein; CD, cluster of differentiation; CTL, cytotoxic T cell; Th, T helper cell; CSE- Conserved sequence elements. This figure was created with the help of the Biorender online tool (https://app.biorender.com/). The figure and description are reproduced with permission from Jawalagatti et al., 2022 (reference 82). ©The American Society of Gene and Cell Therapy.

Despite three decades of history supporting mRNA vaccine development and the successful rollout of mRNA vaccines during the COVID-19 pandemic, the possibility of oral delivery has surprisingly been underexplored (71). This could be attributed to the highly unstable nature of mRNA and the gut posing a significant barrier for mRNA delivery. However, some of the oral antigen delivery strategies such as yeast ghosts, microencapsulated antigens and microbial adhesions have been developed to overcome the harsh conditions in the gut (reviewed in 72). But they suffer from major limitation of poor intestinal epithelial barrier crossing and have not been explored to deliver mRNA (72). Further, lipid-based approaches such as liposomes, bilosomes and immunestimulating complexes (ISCOMs) also provide with a potential delivery vehicle for oral biologic delivery (reviewed in 71). The oral delivery of mRNA vaccines is possible due to the exploitation of an alphaviral replicon and *Salmonella* bactofection for mRNA amplification and gene delivery, respectively. Our group specialized in the development of *Salmonella*-based vaccines against diseases of veterinary and medical importance (73–79), exploited this platform for the development of an oral mRNA vaccine. Further, we exploited the Semliki Forest virus replicon for mRNA amplification (23, 24). We made several modifications to the original vector backbone (pSFV3) to enable transcription in host cells and plasmid maintenance in bacteria (**Figure 1B**) (25). The SP6 promoter was replaced with the Cytomegalovirus (CMV) promoter to enable transcription by mammalian RNA polymerase. The replacement of the ampicillin selection marker with the aspartate-semialdehyde dehydrogenase (asd) auxotrophic marker allows for antibiotic-free plasmid maintenance and delivery (80). The *Salmonella* strains used for gene delivery carry a deletion in the *asd* gene, creating balanced-lethal host-vector systems. Diaminopimelic acid (DAP), the product of asd, is a vital component of the bacterial cell wall, and asd mutants will not survive unless DAP is supplemented in growth media or the *asd* gene is complemented from a plasmid vector. Thus, asd serves as a powerful antibiotic-independent selection maker for bacterial delivery. This DNA-launched-mRNA vector design was exploited for the *Salmonella-*enabled oral delivery of a replicon-based mRNA vaccine against SARS-CoV-2 (25, 81, 82). The detailed mechanism of vector delivery, transgene amplification, and the generation of an immune response upon oral administration of *Salmonella* carrying the SFV replicon vector encoding vaccine immunogens is furnished in **Figure 2**. The findings demonstrate the possibility and potential of bacteria-mediated gene delivery for the development of oral replicon-based mRNA vaccines against infectious diseases.

## Advantages and Limitations of *Salmonella*-Mediated Oral Gene Delivery

The delivery of vaccines through the oral route can elicit a potent mucosal response considering the extensive presence of gut-associated lymphoid tissues (GALT). The bacterial species, *Salmonella* has the ability to interact with immune cells in Payer’s patch, leading to efficient induction of the mucosal response (83, 84). Mucosal vaccines play a pivotal role in limiting infections caused by digestive and respiratory pathogens. Moreover, gut bacteria can influence SIgA production in the lungs through CD103^+^ DCs (85). In agreement, we and others have documented the elicitation of mucosal response in respiratory sites by oral *Salmonella*-based vaccine administration (82, 86). Further, *Salmonella* can translocate through M cells in the intestine and reach organs such as the liver and spleen, eliciting a systemic response as well (87–89). One of the most important advantages of *Salmonella* is its innate ability to invade and proliferate in professional antigen-presenting cells (APCs), such as dendritic cells (DCs) (90) and macrophages (91), during which it directly delivers the DNA cargo to these cells. As antigens must be formed within the APC or cross-presented to an APC to elicit a cellular response (92), gene delivery and antigen expression within APCs result in robust cellular immunity along with the induction of a potent humoral response. Moreover, vaccine production can be easily scaled up, and a high number of doses can be prepared rapidly at an inexpensive rate. Importantly, bacteria-mediated vaccine delivery does not require additional adjuvants or delivery systems, which further cuts down the cost of manufacturing and limits the frequency of vaccine-associated adverse events (68, 69). Most important of all, the availability of licensed oral *Salmonella* Typhi vaccines provides the possibility of direct translation to humans. Further, the availability of a licensed live-attenuated *Vibrio cholerae* vaccine (Vaxchora; https://www.fda.gov/media/98688/download) provides with additional bacterial vector to develop vaccines against diseases of medical importance. The fact that *Salmonella* can be lyophilized permits a thermostable way to dispatch the vaccines and represents progress towards needle-free mass oral immunizations. Collectively, the data suggest the highly prospective nature of exploiting bacteria to develop oral mRNA vaccines with the ability to elicit potent systemic and mucosal immune responses. The intranasal delivery could also be exploited to develop potent mucosal mRNA vaccines. However, as the vaccine uses live-attenuated bacterium poses a significant safety and regulatory hurdle. The intranasal route is more suitable and safer for delivery of mRNA through polymeric delivery systems. The advantage of oral vaccine over an intranasal vaccine would be superior patient compliance and easy mass administration. Therefore, bacteria-mediated delivery of mRNA vaccines for mucosal vaccine development would be feasible when administered orally rather than intranasally.

One of the major limitations of live-attenuated bacteria is safety. However, the availability of tested and proven licensed vaccines provides safer delivery options. Furthermore, well-established tools to modify the bacterial genome provide an opportunity to create safer mutants (93). Another major limitation of using live-attenuated organisms for gene delivery is a hindrance from pre-existing immunity that can seriously affect vaccine efficacy (94, 95). Both SIgA and IgG could contribute to the pre-existing immunity against *Salmonella.* Nevertheless, this limitation could be overcome by deleting the O-antigen ligase (*rfaL*) or any other gene(s) from the bacterial genome that mask the bacteria from detection by the immune system (77). However, several studies have shown the positive influence of pre-existing immunity and recorded stronger immune responses against the delivered antigen by *Salmonella* vectors (reviewed in 96). Thus, the effect of pre-existing immunity on heterologous antigen delivery is likely negligible or less variable. Of note, the effect of pre-existing immunity on viral vectors is more pronounced than on bacterial vectors (96).

## Oral Replicon-Based mRNA Vaccine Against SARS-CoV-2

Our proof-of-principle studies using SARS-CoV-2 (25, 81, 82) provide evidence for the development of oral replicon-based mRNA vaccines against infectious diseases. *Salmonella* is an ideal bacterial vector owing to its unique ability to target GALT upon oral administration, resulting in both systemic and mucosal immune responses in vaccinated individuals. The possibility of oral delivery was partly enabled by creating a DNA-launched-mRNA design of the SFV replicon that essentially drives gene expression by a self-amplifying mRNA mechanism (25). Although the research on RNA vaccines and therapeutics spans over 3 decades, the possibility of oral mRNA vaccine delivery was yet to be explored. To the best of our knowledge, our studies are the first to demonstrate oral replicon-based mRNA vaccine delivery. To this end, we designed a multivalent SFV replicon-based vaccine targeting receptor-binding domain (RBD), heptad repeat domain (HR), membrane glycoprotein (M), and epitopes of nsp13 and employed *Salmonella* Typhimurium for gene delivery (25). The administration of the vaccine was highly safe in mice and hamsters inoculated both orally and intramuscularly (25, 82). The vaccine elicited potent Th1-dominated humoral and cellular immune responses in mice against all the target antigens, suggesting efficient antigen production and presentation (25, 82). Furthermore, RBD expressed after *Salmonella* delivery was confirmed to be antigenically intact in macrophage-like cells (82). We recorded the difference in mucosal immune response induction between oral and intramuscular routes of vaccine administration, highlighting the feasibility of exploiting oral administration for mucosal vaccine development (82). Most importantly, the vaccine protected hamsters against live SARS-CoV-2, and complete protection was elicited by oral immunization against viral replication and lung disease (82). Moreover, a robust cross-protection against the B.1.617.2 delta variant was evidenced following oral immunization in hamsters (82) and mice (81). The fact that an intranasal vaccine durably protected against SARS-CoV-2 variants (97, 98) and dimeric IgA had superior neutralizing activity (99) underscore the efficacy of the mucosal response exerted by oral vaccines in protection against rapidly replicating variants.

## Conclusions and Future Directions

Our proof-of-principle studies have unraveled a novel method for the development of oral mRNA vaccines. The availability of some licensed live-attenuated bacterial vaccines increases the prospects of adopting such vaccines in the clinic. However, more studies using relevant bacterial species in suitable preclinical models are necessary to prove the hypothesis. Moreover, the possibility of other bacterial species, such as *Shigella*, could also be tested to optimize the choice of a bacterial vector.

## Author Contributions

VJ and PK: prepared the figures and wrote the article. JL: acquired funding and commented on the manuscript. All authors contributed to the article and approved the submitted version.

## Funding

This research was supported by Basic Science Research Program through the National Research Foundation of Korea (NRF) funded by the Ministry of Education (2019R1A6A1A03033084).

## Conflict of Interest

The authors declare that the research was conducted in the absence of any commercial or financial relationships that could be construed as a potential conflict of interest.

## Publisher’s Note

All claims expressed in this article are solely those of the authors and do not necessarily represent those of their affiliated organizations, or those of the publisher, the editors and the reviewers. Any product that may be evaluated in this article, or claim that may be made by its manufacturer, is not guaranteed or endorsed by the publisher.
